# Radiomic Features of Hippocampal Subregions in Alzheimer’s Disease and Amnestic Mild Cognitive Impairment

**DOI:** 10.3389/fnagi.2018.00290

**Published:** 2018-09-25

**Authors:** Feng Feng, Pan Wang, Kun Zhao, Bo Zhou, Hongxiang Yao, Qingqing Meng, Lei Wang, Zengqiang Zhang, Yanhui Ding, Luning Wang, Ningyu An, Xi Zhang, Yong Liu

**Affiliations:** ^1^Department of Neurology, Nanlou Division, Chinese PLA General Hospital, National Clinical Research Center for Geriatric Diseases, Beijing, China; ^2^Department of Neurology, The General Hospital of the PLA Rocket Force, Beijing, China; ^3^Department of Neurology, Tianjin Huanhu Hospital, Tianjin, China; ^4^Brainnetome Center, Institute of Automation, Chinese Academy of Sciences, Beijing, China; ^5^School of Information Science and Engineering, Shandong Normal University, Jinan, China; ^6^Department of Radiology, Chinese PLA General Hospital, Beijing, China; ^7^Hainan Branch of Chinese PLA General Hospital, Sanya, China; ^8^National Laboratory of Pattern Recognition, Institute of Automation, Chinese Academy of Sciences, Beijing, China; ^9^CAS Center for Excellence in Brain Science and Intelligence Technology, Institute of Automation, Chinese Academy of Sciences, Beijing, China; ^10^School of Artificial Intelligence, University of Chinese Academy of Sciences, Beijing, China

**Keywords:** alzheimer’s disease, amnestic mild cognitive impairment, hippocampal subregions, radiomic features, support vector machine

## Abstract

Alzheimer’s disease (AD) is characterized by progressive dementia, especially in episodic memory, and amnestic mild cognitive impairment (aMCI) is associated with a high risk of developing AD. Hippocampal atrophy/shape changes are believed to be the most robust magnetic resonance imaging (MRI) markers for AD and aMCI. Radiomics, a method of texture analysis, can quantitatively examine a large set of features and has previously been successfully applied to evaluate imaging biomarkers for AD. To test whether radiomic features in the hippocampus can be employed for early classification of AD and aMCI, 1692 features from the caudal and head parts of the bilateral hippocampus were extracted from 38 AD patients, 33 aMCI patients and 45 normal controls (NCs). One way analysis of variance (ANOVA) showed that 111 features exhibited statistically significant group differences (*P* < 0.01, Bonferroni corrected). Among these features, 98 were significantly correlated with Mini-Mental State Examination (MMSE) scores in AD and aMCI subjects (*P* < 0.01). The support vector machine (SVM) model demonstrated that radiomic features allowed us to distinguish AD from NC with an accuracy of 86.75% (specificity = 88.89% and sensitivity = 84.21%) and an area under curve (AUC) of 0.93. In conclusion, these findings provide evidence showing that radiomic features are beneficial in detecting early cognitive decline, and SVM classification analysis provides encouraging evidence for using hippocampal radiomic features as a potential biomarker for clinical applications in AD.

## Introduction

As the leading cause of neurodegenerative dementia, Alzheimer’s disease (AD) is characterized by a progressive deterioration in cognitive function, especially in episodic memory. Due to its influence on the normal lives of both patients and caregivers, AD has become a considerable burden on society (Reitz and Mayeux, 2014; Alzheimer’s Association, 2017). Mild cognitive impairment (MCI) is generally defined as a transitional stage between the cognitive changes associated with normal aging and early dementia. Amnestic MCI (aMCI) means that while the memory ability of a patient has decreased, the patient does not fulfill the criteria for dementia, and aMCI is thought to be the prodromal stage of dementia due to AD and has a high risk of developing into AD (Petersen and Jack, 2009; Petersen, 2016).

Medial temporal atrophy is believed to be one of the magnetic resonance imaging (MRI) markers for progression to AD in a prodromal stage, and atrophy of the hippocampus, the most vulnerable structure in the medial temporal lobe, is one of its most robust markers (Ferreira et al., 2011; Yang et al., 2012; Ezzati et al., 2016). In the past decade, structural MRI has been widely used to quantify hippocampal atrophy for distinguishing MCI from AD (Pruessner et al., 2000; Shen et al., 2002; Apostolova et al., 2012). Evidence has also demonstrated that aMCI patients converting to AD show greater atrophy in the hippocampus than is found in those who do not convert to AD (Chételat et al., 2005; Apostolova et al., 2006; Leung et al., 2013). Beyond the decrease in volume, changes in the morphology of the hippocampus have also been found and may appear even earlier than atrophy in AD (Achterberg et al., 2014; Sørensen et al., 2016).

Radiomics, a morphological method for imaging analysis, can quantitatively examine a large set of texture features (Parmar et al., 2015) and is used in the classification of tumors and in predicting radiation therapy outcomes (Huynh et al., 2016). The implications of “texture” include many image properties, such as coarseness, rugosity, and smoothness. Recently, texture analysis has been successfully applied to produce imaging biomarkers for AD (de Oliveira et al., 2011; Anandh et al., 2015; Chincarini et al., 2015). For example, studies have shown that hippocampal texture abnormalities appear in MCI/AD, indicating that texture may serve as a prognostic neuroimaging biomarker for early cognitive impairment (Sørensen et al., 2016, 2017).

Hippocampal head (anterior) atrophy is the most obvious in AD (Raji et al., 2009) and has been reported as a predictive marker of conversion to AD (Costafreda et al., 2011). Convergence evidence has also demonstrated the existence of functional differences along the anterior-posterior axis of the hippocampus (Strange et al., 2014; Collin et al., 2015). The anterior-posterior discrepancies in the hippocampus have also been associated with neuropsychiatric symptoms in early AD (Lyketsos et al., 2011). Given that the posterior and anterior parts of the hippocampal are differentially vulnerable to neuropathology in AD (La Joie et al., 2014; Delli Pizzi et al., 2016; Zeidman and Maguire, 2016), evaluating imaging measurements in different subfields would provide more accurate and sensitive information for the early detection of AD (La Joie et al., 2013; Blanken et al., 2017; Mak et al., 2017). Thus, hippocampal morphological differentiation along the posterior-anterior axis of the hippocampus deserves more attention because they are relevant for the early diagnosis of AD.

**FIGURE 1:**
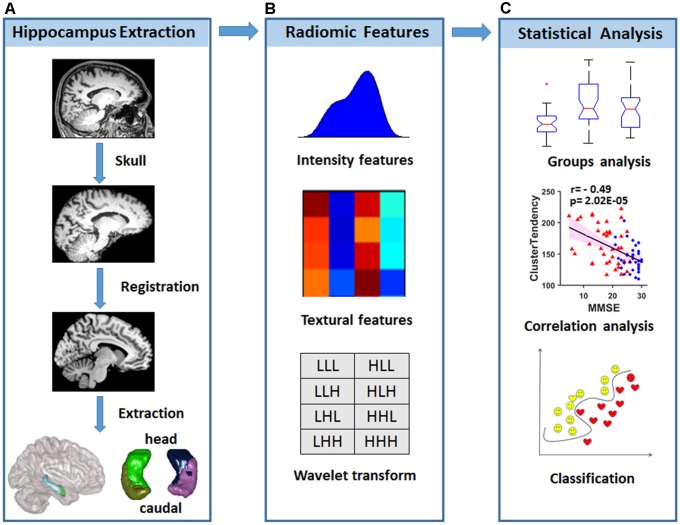
Diagram of data processing and statistical analyses. **(A)** Strategy for hippocampal subregion extraction from high-resolution structural MRI. **(B)** Intensity features and textural features were extracted from the images, and wavelet transformation was achieved in each hippocampal subregion. **(C)** Statistical analysis was used to find radiomic features that were different among the groups, and correlations with the MMSE were performed to evaluate the relationship between radiomic features and cognitive performance. A classification analysis was performed to determine whether radiomic features could be used for diagnosis.

Inspired by the above studies, we hypothesized that radiomic features in the hippocampus would be disrupted and that these changes might be employed in the early classification of AD and aMCI. To test this hypothesis, radiomic features that were used in previous studies (Aerts et al., 2014; Parmar et al., 2015) were calculated from hippocampal subregions based on the Brainnetome Atlas (Fan et al., 2016) from a structural MRI data of 38 AD patients, 33 aMCI patients and 45 normal controls (NCs). One-way ANOVA was then used to identify changes in the radiomic features among AD, aMCI and NC subjects. Then, correlation analyses between the identified radiomic features and Mini-Mental State Examination (MMSE) scores were calculated to evaluate the relationships between hippocampal textures and cognitive ability. In addition to these case-control comparisons, we employed the support vector machine (SVM) model to evaluate the diagnostic power of radiomic features (**Figure 1**).

## Materials and Methods

### Ethics Statement, Subject Recruitment, and Neuropsychological Assessment

This study was approved by the Medical Ethics Committee of the Chinese PLA General Hospital. All subjects or their legal guardians (a family member) signed written informed consent forms. All subjects met identical methodological stringency criteria, and comprehensive clinical details can be found elsewhere in our previous studies (Zhang Z. et al., 2012, 2014; Yao et al., 2013; Zhou et al., 2013; Wang et al., 2015). To maintain the scientific integrity of the present study, herein we provide a brief introduction regarding the data inclusion and exclusion criteria, acquisition and processing.

The recruited AD patients fulfilled the NINCDS-ADRDA (National Institute of Neurological and Communicative Disorders and Stroke and the AD and Related Disorders Association) criteria for the diagnosis of probable AD (McKhann et al., 1984). The aMCI patients were required to conform to the criteria described by Petersen (Petersen, 2004). The AD and aMCI patients also met the core clinical criteria of the new diagnostic criteria for probable AD and aMCI due to AD (Albert et al., 2011; McKhann et al., 2011). The NCs included subjects lacking memory decline but matching with AD and aMCI patients in gender and age. All of the participants were 55 to 85 years old and were neurological inpatients and outpatients of the Chinese PLA General Hospital. Clinical, physical and neuropsychological assessments were performed before MRI examination. Subjects with neurological or psychiatric diseases or with a history of cerebrovascular attacks or other degenerative disorders were excluded.

All subjects underwent a battery of neuropsychological tests at the department of neurology of the Chinese PLA General Hospital. These tests included the MMSE, Clinical Dementia Rating, Auditory Verbal Learning Test (AVLT), Geriatric Depression Scale and Activities of Daily Living scale.

### Structural MRI Data Acquisition

#### Discovery Data

Magnetic resonance imaging examinations were performed at the department of radiology of the Chinese PLA General Hospital using a 3.0 T Siemens MR system (Skyra, Siemens, Germany) with a 20-channel head coil. During the examinations, the subjects were given comfortable foam padding to minimize head motion and ear plugs to reduce the scanner noise. Before the structural MRI data were collected, T2-weighted images were collected and evaluated by two senior radiologists. Sagittal T1-weighted structural images (192 continuous slices) were acquired for each subject using a magnetization-prepared rapid gradient echo sequence with the following scan parameters: repetition time (TR) = 2,530 ms, echo time (TE) = 3.43 ms, inversion time (TI) = 1100 ms, field of view (FOV) = 256 mm × 256 mm, acquisition matrix = 256 × 256, flip angle (FA) = 7°, and slice thickness = 1 mm. The obtained three-dimensional images had a resolution of 1 mm × 1 mm × 1 mm.

#### Replicated Data

Magnetic resonance imaging examinations were further performed in the same department of radiology using another 3.0 T Siemens MR system (Skyra, Siemens, Germany) for other subjects, including AD and aMCI patients and NCs. The protocol and parameters used in the MRI examinations were all consistent with those used in the discovery data.

### Hippocampal Radiomic Feature Extraction

All preprocessing steps were performed using statistical parametric mapping (SPM12)^1^ and the Brainnetome fMRI Toolkit^2^ (Xu et al., 2018). Briefly, each individual T1-weighted DICOM image of the brain was first converted to NIFTI data. Next, skull stripping was performed, and the obtained images were normalized to the Montreal Neurological Institute (MNI) standard T1 template (standard space 181 × 217 × 181 with a resolution of 1 mm × 1 mm × 1 mm). Meanwhile, we resliced the Brainnetome Atlas^3^ to the standard MNI space with a resolution of 1 mm × 1 mm × 1 mm, and the caudal and head regions of the bilateral hippocampus were further extracted as masks. Lastly, for each subject, we obtained the subregions by point multiplication of the masks and the normalized T1 images.

Quantitative radiomic features were calculated using in-house MATLAB script as previously reported (Aerts et al., 2014; Huynh et al., 2016), and detailed descriptions of each feature are listed in the supplemental information to maintain the scientific integrity of the present study. Briefly, the intensity features were calculated based on the histogram, which represented the distribution of voxel intensities within the images (14 features), the textural features on the gray level co-occurrence matrix (GLCM) and the gray level run-length matrix (GLRLM) (33 features). Wavelet transformation (Symlet wavelet filter, “sym4” were used here) in eight directions (LLL, LLH, LHL, LHH, HLL, HLH, HHL, and HHH) was performed for the four hippocampal subregions to combine the spatial and frequency characteristics (for more detailed information, please refer to the **Supplementary Material**). As a result, 423 radiomic features were obtained for each hippocampal subregion, resulting in the inclusion of a total of 1692 (423 × 4) features for further analyses (**Figure 1**).

### Statistical Analysis

Radiomic features were adjusted using the linear regression method to control the age and gender effects and were then statistically tested to determine the number of indices with significant differences among groups and to perform the further correlation analysis.

One-way ANOVA was employed to evaluate the differences between the AD, aMCI and NC groups at each subregion. Hence, 423 × 4-fold comparisons were performed, and we then used the Bonferroni correction to control type 1 error with *P* < 0.01/(*N* = 423 × 4). A *post hoc* analysis was performed to verify the differences between any two groups.

To assess the association between radiomic texture and cognitive ability, Spearman’s correlation coefficient was calculated to evaluate the relationships between the identified features and MMSE (*P* < 0.01). In addition, a detailed map was provided of the correlations between these features and the AVLT scores in immediate recall, delayed recall, recognition of primary and new words (*P* < 0.05). To evaluate the replication of the results related to radiomic features that showed significant differences and their correlations with MMSE, another dataset was further analyzed using the same statistical methods.

### Classification Analysis

To assess the multivariate performance of radiomic features, a classification model was established based on the SVM. A nonlinear SVM with a radial basis function (RBF) kernel was employed in LIBSVM.^4^ The performance of the classifier was evaluated by the leave-one-out cross-validation (LOOCV) method, which has been widely used as a reliable estimating approach of true generalization performance. In the present study, the following univariate feature-ranking approach based on *t*-test was performed, as described in previous studies (Kamkar et al., 2015; Beheshti et al., 2016):

$$T=\frac{\mu_{c1}-\mu_{c2}}{\sqrt{\frac{\sigma_{c1}^{2}}{\eta_{c1}}}+\sqrt{\frac{\sigma_{c2}^{2}}{\eta_{c2}}}}$$

where *μ_ck_* σ^2^*_ck_* and *n_ck_* (*k* = 1,2) are the mean, variance, and number of samples, respectively, in the two classes (c1 and c2: AD and NC). Each feature was normalized before the rank analysis was performed with the following method:

$$X=\frac{X_{i}-X_{\min}}{X_{\max}-X_{\min}}(i=1.....N)$$

These features were ranked by the *t*-test, with a high value indicating large discrimination performance in the training dataset. To avoid over-filtering, only up to 200 features were selected for analysis.

In this model, there were two LOOCV procedures for grid search (Beheshti et al., 2016). In the inner and outer loop, there were both training data and testing data. First, the feature rankings with *t*-tests were employed in the inner loop’s training data; the parameters c (regularization) and g (control the kernel width) obtained by LOOCV and grid search were also used. Then, the feature rankings with *t*-tests were further employed in the outer loop’s training data. Lastly, the parameters c and g obtained from the inner loop’s training data were used in the testing data to access the classification performance.

Input: radiomic features
  for i ← 1 to *N* (the number of data)
  do: testing data = data (i)
  training data = data (*N*| i)
  Ranking features with *t*-test among training data
  for j ← 1 to *N*-1
  do: LOOCV with different c and g.
  Compute the accuracy for each c and g.
  Choose the best c and g.
  Compute the accuracy for classification.
  Output: the best classification results

The classification performance was evaluated by means of accuracy (ACC), sensitivity (SEN), and specificity (SPE). The diagnostic capabilities of the radiomic features were evaluated using receiver operating characteristic (ROC) curves with the corresponding area under the ROC curve (AUC). Finally, to assess the clinical relevance of this radiomic-based classification, we investigated correlations between the classifier output and cognitive ability scores (MMSE) in individual subjects.

### Data Sharing

The patients’ hippocampus nii image files, the texture features and the codes are available online at https://github.com/yongliulab.

## Results

### Demographic Characteristics and Neuropsychological Assessment of Groups

In the present study, 116 subjects (38 AD patients, 33 aMCI patients and 45 NCs) were included in the discovery data. Mean age and gender ratio had no significant differences (*P* > 0.05). The MMSE score was remarkably and significantly different (*P* < 0.001), with AD patients having the lowest scores and subjects in the NC group having the highest scores. For the scores on the AVLT in immediate recall, delayed recall, and recognition of primary words and new words, the same sequence was observed among AD, aMCI and NC subjects (*P* < 0.001) (**Table 1**). The replicated dataset, which included 42 AD patients, 37 aMCI patients and 43 age- and gender-matched NCs, was used for a replication analysis of the identified feature changes (**Supplementary Table S1**).

**Table 1:** Demographic, clinical and neuropsychological discovery data for AD, aMCI, and NC subjects.

	NC (*n* = 45)	aMCI (*n* = 33)	AD (*n* = 38)	*P*
Age (years)	68.2 ± 6.9	70.6 ± 8.2	71.7 ± 8.3	0.102
Gender (M/F)	22/23	14/19	16/22	0.680
MMSE score	28.6 ± 1.4	26.6 ± 2.6^a^	17.6 ± 5.6^a,b^	<0.001
AVLT-Immediate Recall^c,e^	5.6 ± 1.2	4.2 ± 1.4^a^	3.0 ± 1.3^a,b^	<0.001
AVLT-Delayed Recall^d,e^	5.6 ± 1.9	2.5 ± 2.3^a^	0.6 ± 1.1^a,b^	<0.001
AVLT-Recognition (primary words)^e^	9.4 ± 1.1	8.4 ± 1.5^a^	6.2 ± 3.5^a,b^	<0.001
AVLT-Recognition (new words)^e^	9.8 ± 0.7	8.7 ± 2.1^a^	6.8 ± 3.2^a,b^	<0.001

*A Chi-square test was used for gender comparisons, and ANOVA with Bonferroni’s post hoc test was used for age and neuropsychological test comparisons. ^a^Significant compared with NC.^b^Significant compared with aMCI. ^c^The mean of three scores for every immediate recall. ^d^The scores for delayed recall after five minutes. ^e^Thirteen AD subjects, 2 aMCI subjects and 3 NC subjects could not or refused to complete this test. MMSE, Mini-Mental State Examination; AVLT, auditory verbal learning test.*

### Radiomic Features in AD, aMCI and NC Subjects

**FIGURE 2:**
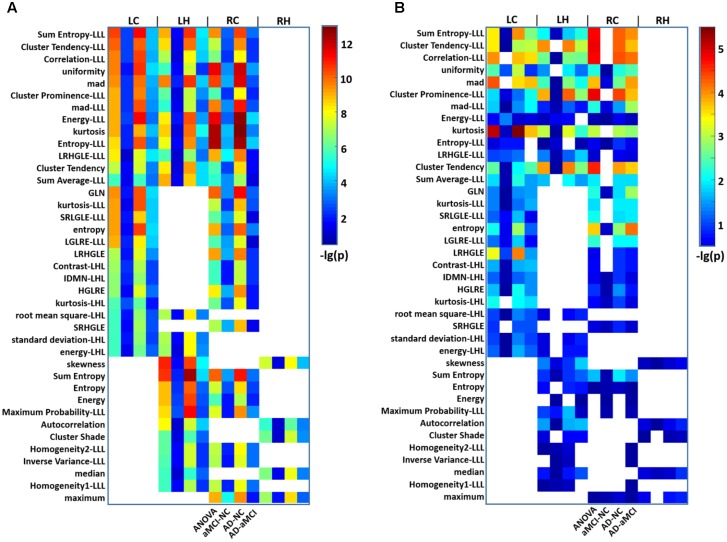
Heat maps of radiomic features that were significantly different among groups in more than one hippocampal subregion in AD and aMCI subjects. **(A)** A total of 39 radiomic features showed significant differences among groups in more than one hippocampal subregion. Thirteen features were identified in the LC, LH, and RC subregions at the same time. For each feature observed in four subregions, there were four grids (*P*-values for ANOVA and *P*-values for the *t*-tests between aMCI and NC, AD and NC, and AD and aMCI). The color bar represents the –lg(*P*) values, a blank grid indicates there was no significant alteration in the related radiomic feature between AD and aMCI or AD and NC subjects (*P* < 0.01, Bonferroni corrected). **(B)** The replication results of another dataset were analyzed using the same procedure. LC, Left caudal; LH, Left head; RC, Right caudal; RH, Right head; mad, mean absolute deviation; LRHGLE, Long run, high gray level emphasis; GLN, Gray level non-uniformity; SRLGLE, Short run, low gray level emphasis; LGLRE, Low gray level run emphasis; IDMN, Inverse difference moment normalized; HGLRE, High gray level run emphasis; SRHGLE, Short run, high gray level emphasis.

As a whole, 111 features showed significant differences (*P* < 0.01, Bonferroni corrected with *N* = 423 × 4) among the three groups. Of these, 39 parameters were altered in more than one subregion (**Figure 2A**). The *post hoc* analysis further demonstrated that differences were more significant when comparing the AD and NC groups than that when comparing the AD and aMCI groups or the aMCI and NC groups (**Figure 2A**). Considering the category of these features, 8 of the 39 parameters belonged to the intensity features, 13 were textural features of the GLCM, 6 were from the GLRLM (**Table 2**). As shown in **Figure 2A**, most of the 39 parameters were found in the left caudal, left head and right caudal regions of the hippocampus, whereas a small number of parameters were found in the right head region of the hippocampus. The features that indicated the most significant alterations among groups included kurtosis (-lg(*P*) = 12.97), Energy-LLL (-lg(*P*) = 12.60) and Entropy-LLL (-lg(*P*) = 12.54) in the right caudal part and Sum Entropy (-lg(*P*) = 12.57) in the left head part (**Figure 2A**). The same 39 parameters were evaluated in the replication dataset, in which they showed similar but weaker alterations (**Figure 2B**). Note that the most significant changes were identified in the caudal part of right hippocampus in the AD group in both datasets (**Figure 2**).

**Table 2:** Summary of radiomic features with significant differences in hippocampal subregions.

Type of features	Detailed features
Intensity features (8/14)	uniformity, mad, kurtosis, entropy, root mean square, standard deviation, energy, skewness
Textural features of GLCM (13/22)	Sum Entropy, Cluster Tendency, Correlation, Cluster Prominence, Energy, Entropy, Sum Average, Contrast, IDMN, Maximum Probability, Autocorrelation, Cluster Shade, Homogeneity
Textural features of GLRLM (6/11)	GLN, LRHGLE, SRLGLE, LGLRE, HGLRE, SRHGLE

*GLCM, Gray-Level Co-Occurrence Matrix; GLRLM, Gray-Level Run-Length Matrix; mad, mean absolute deviation; IDMN, Inverse difference moment normalized; GLN, Gray level non-uniformity; LRHGLE, long run, high gray level emphasis; SRLGLE, short run, low gray level emphasis; LGLRE, low gray level run emphasis; HGLRE, high gray level run emphasis; SRHGLE, short run, high gray level emphasis.*

### Correlations Between Radiomic Features and MMSE Scores

**FIGURE 3:**
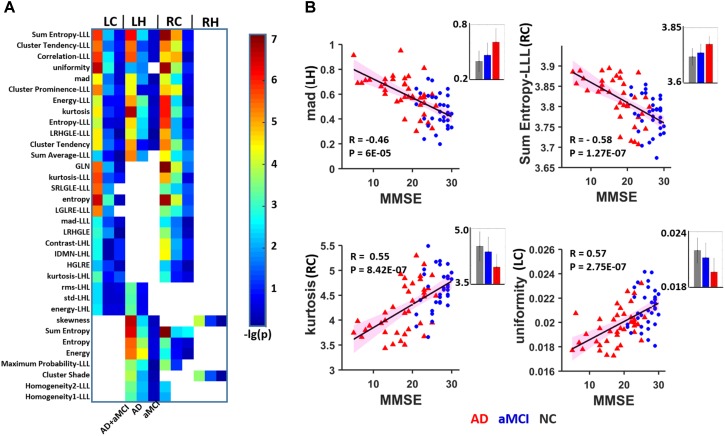
Heat map of radiomic features correlating with MMSE in more than one hippocampal subregion. **(A)** A total of 16 features were significantly correlated with the MMSE in two or three subregions (*P* < 0.01, Bonferroni corrected). The color bar represents the –lg(*P*) values, a blank grid indicates there was no significant correlation between the related radiomic features and MMSE scores between AD and aMCI. **(B)** Four typical features were selected for illustration in scatter diagrams. The corresponding mean values are provided (gray: NC, blue: aMCI; red: AD). LC, Left caudal; LH, Left head; RC, Right caudal; mad, mean absolute deviation; LRHGLE, Long run, high gray level emphasis; GLN, Gray level non-uniformity; SRLGLE, Short run, low gray level emphasis; LGLRE, Low gray level run emphasis; IDMN, Inverse difference moment normalized; HGLRE, High gray level run emphasis.

A total of 98 among the 111 identified features were significantly correlated with MMSE scores in AD and aMCI subjects (*P* < 0.01). Among these, 34 parameters in the bilateral subregions of the hippocampus were significantly correlated in two or three subregions (**Figure 3A** and **Table 3**). The other 64 features in one subregion are shown in **Supplementary Figure S2**. To construct a sketch map of these correlations, four typical features were selected for illustration in scatter diagrams (**Figure 3B**). Importantly, the same 34 parameters were significantly correlated with MMSE in more than one subregion in the replication dataset (*P* < 0.05, uncorrected) (**Supplementary Figure S3B**). For the bilateral caudal and left head subregions, approximately 50 parameters were significantly correlated with AVLT scores in more than one subregion, while 4 textural features were significantly correlated with AVLT scores in the right head region (**Supplementary Figure S4**).

**Table 3:** Summary of radiomic features correlated with MMSE scores in hippocampal subregions.

Type of features	Detailed features
Intensity features (8/14)	uniformity, mad, kurtosis, entropy, energy, root mean square, standard deviation, skewness
Textural features of GLCM (13/22)	Sum Entropy, Cluster Tendency, Correlation, Cluster Prominence, Entropy, Energy, IDMN, Sum Average, Contrast, Maximum Probability, Cluster Shade, Homogeneity2, Homogeneity1
Textural features of GLRLM (5/11)	LRHGLE, GLN, SRLGLE, LGLRE, HGLRE

*GLCM, Gray-Level Co-Occurrence Matrix; GLRLM, Gray-Level Run-Length Matrix; mad, mean absolute deviation; IDMN, inverse difference moment normalized; LRHGLE, Long run, high gray level emphasis; GLN, Gray level non-uniformity; SRLGLE, short run, low gray level emphasis; LGLRE, low gray level run emphasis; HGLRE, high gray level run emphasis.*

### Classification Performance

**FIGURE 4:**
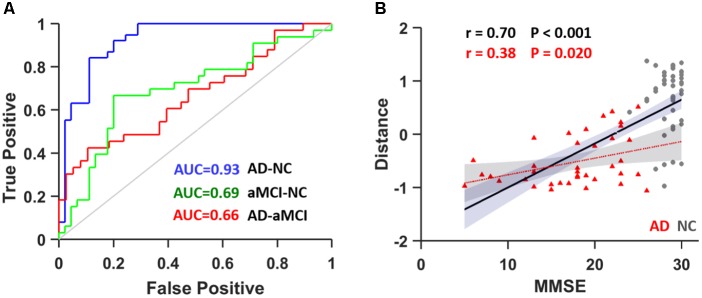
**(A)** ROC curve for distinguishing between AD and NC subjects (blue), aMCI and NC subjects (green) and AD and aMCI subjects (red). **(B)** The correlation between the distance from the hyperplane and MMSE scores in AD plus NCs (black line) as well as in AD (red line). AUC, area under curve.

We introduced the SVM model to determine whether these textures were good features for classification analysis in the discovery data (*N* = 116, 38 AD patients, 33 aMCI patients and 45 NCs). To avoid over-filtering, up to 200 top features based on *t*-test rankings were selected for classification analysis. After training steps, we obtained the maximum classification accuracy with 163 features. **Table 4** presents the classifier performance results obtained using LOOCV for SVM classifiers in terms of ACC, SEN, SPE, and AUC. The accuracy of the feature vectors with 163 features for SVM classifiers was 86.75% (SPE = 88.89%, SEN = 84.21%, and AUC = 0.93) for distinguishing AD from NC. This rate was reduced by approximately 10% for all performance indicators (ACC, SEN, SPE, and AUC) when using the same features to distinguish aMCI from NC or AD from aMCI (**Figure 4**). In addition, we found that the distance from the hyperplane was highly correlated with MMSE scores in the AD plus NC (r = 0.70, *P* < 0.001) and AD (r = 0.38, *P* = 0.020) groups designated by the classification analysis (**Figure 4B**).

**Table 4:** Classification performance by the classification features (feature number = 163) between two groups.

	AD-NC	aMCI-NC	AD-aMCI
ACC	86.75%	70.51%	59.15%
SPE	88.89%	80.00%	63.16%
SEN	84.21%	57.58%	54.55%

*The detailed receiver operating characteristic curve (ROC) plot can be found in **Figure 4**. ACC, accuracy; SPE, specificity; SEN, sensitivity.*

## Discussion

In the present study, we are the first to identify significantly different radiomic features in hippocampal subregions among AD and aMCI patients and NCs. These features demonstrated were significantly associated with cognitive ability in AD and/or aMCI subjects. More importantly, an SVM analysis demonstrated that these hippocampal textures are potential markers of AD.

In recent years, MRI-based biomarkers of AD that target gray matter atrophy or shape were found to be the most commonly used measures (Risacher et al., 2009; Ezzati et al., 2016; Caldwell et al., 2017). Reduced hippocampal volume has been well studied in AD and MCI individuals (for a review, see Shi et al., 2009). In the revised National Institute on Aging-Alzheimer’s Association diagnostic criteria, hippocampal atrophy was one of the core markers for AD (Albert et al., 2011; Catani et al., 2013). Except for the reduced volume, abnormal metabolism levels, disrupted brain activity and microstructural properties within the hippocampus have been well reported (for a review, see Huijbers et al., 2015). The current results show that radiomic features are different in more than one subregion of the hippocampus, especially in the caudal parts. Among these identified to show altered texture features, three indices (kurtosis, Energy-LLL and Entropy-LLL) from the right caudal region exhibited the most significant alterations. In probability theory and statistics, kurtosis is a measure of flatness in probability distributions in brain images.^5^ Entropy is a statistical measure of randomness that can be used to characterize the texture of an image. Significant differences in the above features indicate that cognitive impairments in AD and aMCI might result in complicated and changed distributions of voxel values within the hippocampus. This inference was supported by the significant correlation found between texture features and MMSE scores in the AD/aMCI groups and has also been confirmed by previous related studies (de Oliveira et al., 2011; Sørensen et al., 2016). Several investigators have reported that the shapes of brain structures can provide an additional dimension (structural morphology) when quantifying alterations in cognitive ability in AD/MCI patients (Qiu et al., 2008; Tang et al., 2015, 2016). However, hippocampal shape abnormalities (Apostolova et al., 2006; Christensen et al., 2015) and texture features (Li et al., 2010; Zhang J. et al., 2012) have been reported to be associated with individual memory performance. Hence, the present study provides additional clues regarding the morphological alterations that occur in the hippocampus in AD.

As shown in the present study, several texture measures (such as the mean absolute deviation (mad), kurtosis, uniformity, entropy and Sum Entropy-LLL) showed a significant correlation with the MMSE score in AD/aMCI. Among these measures, mad reflects dispersion, kurtosis represents flatness, and both uniformity and entropy measure randomness of the intensity value distribution. For example, a positive kurtosis indicates a more peaked histogram than a Gaussian (normal) distribution of the selected image in AD than that in NC. This phenomenon might be caused by the atrophy of gray matter, which results in a less highly peaked histogram for the voxel intensity values similar to that due to atrophy. It should also be noted that the MMSE is limited in terms of its sensitivity to high and low levels of cognitive functioning (Tombaugh and McIntyre, 1992). Moreover, the correlation results between the textures and the AVLT scores immediate recall, delayed recall and recognition of primary words and new words provide further evidence suggesting that texture is an important and beneficial supplementary index, in addition to volume measurements, for understanding impaired cognitive ability in patients. Although the pathological features of AD, such as neurofibrillary tangles (NFTs) and amyloid-β (Aβ) plaques, cannot be detected on MRI, these microstructural changes might lead to altered textural patterns (Castellano et al., 2004) and might be manifested by texture analysis (Csernansky et al., 2005; Manning et al., 2015; Hwang et al., 2016). Indeed, a negative correlation between image textures and FDG-PET metabolism was identified in a recent excellent study (Shi et al., 2009). Regions with the highest atrophy rates were demonstrated to be located in the anterolateral hippocampus, which is also the region with the highest tau deposition (Franko and Joly, 2013). The anterior (head) hippocampus is thus thought to be the brain region in which the majority of volume differences are found in aMCI and AD patients.

Despite the identification of significantly different features and their correlation with MMSE scores, the application of these hippocampal features as a biomarker still needs to be confirmed. Using simple and common models of nonlinear (RBF) SVM, we obtained an accuracy of 86.75% (SPE: 88.89%; SEN: 84.21%) for distinguishing AD from NC by LOOCV. This finding is consistent with many previous imaging studies (Mak et al., 2011; Zhang Z. et al., 2012; Guo et al., 2014) suggesting that the hippocampal textures might be potential imaging markers for AD. The performance in terms of ACC, SEN, SPE, and AUC was competitive with several state-of-the-art results reported in previous studies (Jie et al., 2015; Ritter et al., 2015; Beheshti et al., 2016; Schouten et al., 2016). In addition, by analyzing the frequencies of the features selected in each LOOCV run, we found that the most powerful feature was the “intensity” of the hippocampus. More importantly, the individualized distance to the hyperplane was a neuroanatomical signature of AD and was significantly correlated with MMSE scores, indicating that the more severe AD becomes, the more likely it is to be identified using radiomic features based on the classifier’s output. This result means that the atrophy and shape of the hippocampus play a very important role in distinguishing AD patients from healthy controls, as suggested by previous studies (Hampel et al., 2008; Beheshti et al., 2016; Sørensen et al., 2016).

## Limitations

There were several limitations in this study. First, the hippocampal subregions segmentation was based on the atlas, which resulted in the same shape features in different subregions and, thus, were excluded from the analysis. Because only the hippocampus was included, some other important regions, such as the parahippocampus, amygdala and ventricles, should be investigated in future work. The hippocampus is also divided into subfields, including the Cornu Ammonis (CA1–4), the dentate gyrus and the subiculum, each of which has distinctive histological characteristics and specialized functions (Bird and Burgess, 2008; Delli Pizzi et al., 2016; Zeidman and Maguire, 2016; Blanken et al., 2017). However, radiomic feature extractions are unsuitable for such small regions as the above-described parcellation. Second, the results of the present study demonstrate that radiomic features have potential use in clinical diagnosis; meanwhile, we should also admit that the LOOCV might also have potentially overestimating performance (Varoquaux, 2017; Varoquaux et al., 2017). To validate if the result was robust, we performed a leave-four-subjects-out cross-validation and simulation 1000 times, and the results showed that we could obtain an accuracy of 83.55% (SPE: 84.66%; SEN: 82.66%) for distinguishing AD from NC. Using larger multi-center datasets is a solution to future challenges in reproducibility and statistical power by taking testing data from independent centers. Furthermore, a prospective longitudinal study with a large multi-center sample size is needed to detect the earlier stages of AD (Dubois et al., 2016). Lastly, although we believe this approach will increase our understanding of the multiple levels of hippocampal alterations observed over the course of AD, the radiomic features is much less widespread and less well developed than are other imaging approaches, therefore, combining texture with other markers (for example brain volume, cortical thickness, functional connectivity, and CSF, etc.) to achieve a powerful biomarker of sufficient quality to be considered for clinical applications is needed for future studies.

## Conclusion

In conclusion, in the present study, we found that hippocampal radiomic features exhibit significant disease-severity-related alterations in AD. We specifically investigated hippocampal textures as an MRI-based biomarker of AD. The results showed that hippocampal textures could be used as potential MRI markers for the early detection of AD from NC, with a relatively high correction ratio (ACC = 86.75%, specificity = 88.89%, and sensitivity = 84.21%) with the LOOCV method. The results of the present study highlight the importance of hippocampal texture abnormalities in AD and support the possibility that textures may serve as a neuroimaging biomarker for the early detection of AD and aMCI.

## Author Contributions

FF, PW, BZ, HY, QM, ZZ, LNW, LW, and NA collected the data. YL, KZ, and YD analyzed the data and performed the measurements. FF, KZ, and YL had the major responsibility of preparing the paper. YL and XZ supervised the project.

## Conflict of Interest Statement

The authors declare that the research was conducted in the absence of any commercial or financial relationships that could be construed as a potential conflict of interest.

## Footnotes

1 http://www.fil.ion.ucl.ac.uk/spm

2 http://brant.brainnetome.org

3 http://atlas.brainnetome.org

4 http://www.csie.ntu.edu.tw/tildecjlin/libsvm/

5 https://en.wikipedia.org/wiki/Kurtosis
