# Supplementary material for: Radiomic Features of Hippocampal Subregions in Alzheimer’s Disease and Amnestic Mild Cognitive Impairment
Source: Front Aging Neurosci. 2018 Sep 25;10:290. doi: 10.3389/fnagi.2018.00290 (PMC6167420; doi:10.3389/fnagi.2018.00290)
Supplement: Supplementary file 1 [file Data_Sheet_1.docx]

Supplementary Information

Outline

[Supplementary Tables 2](#_Toc520295205)

[Supplementary Figures 3](#_Toc520295206)

[Figure S1 3](#_Toc520295207)

[Figure S1 4](#_Toc520295208)

[Figure S3 5](#_Toc520295209)

[Figure S4 6](#_Toc520295210)

[Supplementary Methods 7](#_Toc520295211)

[Cross-validation with leave-four-out for classification AD from NC 7](#_Toc520295212)

[Radiomics features 8](#_Toc520295213)

[1. Intensity features 8](#_Toc520295214)

[2. Textural features 9](#_Toc520295215)

[3. Wavelet features 13](#_Toc520295216)

[References 14](#_Toc520295217)

# Supplementary Tables

**Table S1** Demographic, clinical and neuropsychological data from AD, aMCI and NC subjects of the replicated data.

|  | NC (n = 43) | aMCI (n = 37) | AD (n = 42) | P |
| --- | --- | --- | --- | --- |
| Age (years) | 68.3±8.0 | 69.8±6.9 | 70.4±8.7 | 0.449 |
| Gender (M/F) | 20/23 | 13/24 | 16/26 | 0.553 |
| MMSE score | 28.8±1.2 | 26.3±2.6 ^a^ | 15.8±5.7 ^a,b^ | 0.000 |
| AVLT-Immediate Recall ^c,e^ | 6.3±1.0 | 4.9±1.3 ^a^ | 3.1±1.4 ^a,b^ | 0.000 |
| AVLT-Delayed Recall ^d,e^ | 6.3±2.0 | 4.1±2.2 ^a^ | 0.4±1.3 ^a,b^ | 0.000 |
| AVLT-Recognition (primary words) ^e^ | 9.4±1.2 | 8.8±1.9 ^a^ | 6.2±3.4 ^a,b^ | 0.000 |
| AVLT-Recognition (new words) ^e^ | 9.8±0.5 | 9.4±1.8 ^a^ | 6.3±3.4 ^a,b^ | 0.000 |

A Chi-square test was used for gender comparisons, and ANOVA was used for age and neuropsychological test comparisons.

^a^ Significant compared with NC. ^b^ Significant compared with aMCI.^c^ The mean of three scores for every immediate recall. ^d^ The scores for delayed recall after five minutes. ^e^ Fourteen AD subjects, 3 aMCI subjects and 3 NC subjects could not or refused to complete this test.

Abbreviations: MMSE, Mini-Mental State Examination; AVLT, auditory verbal learning test

# Supplementary Figures

## Figure S1

**
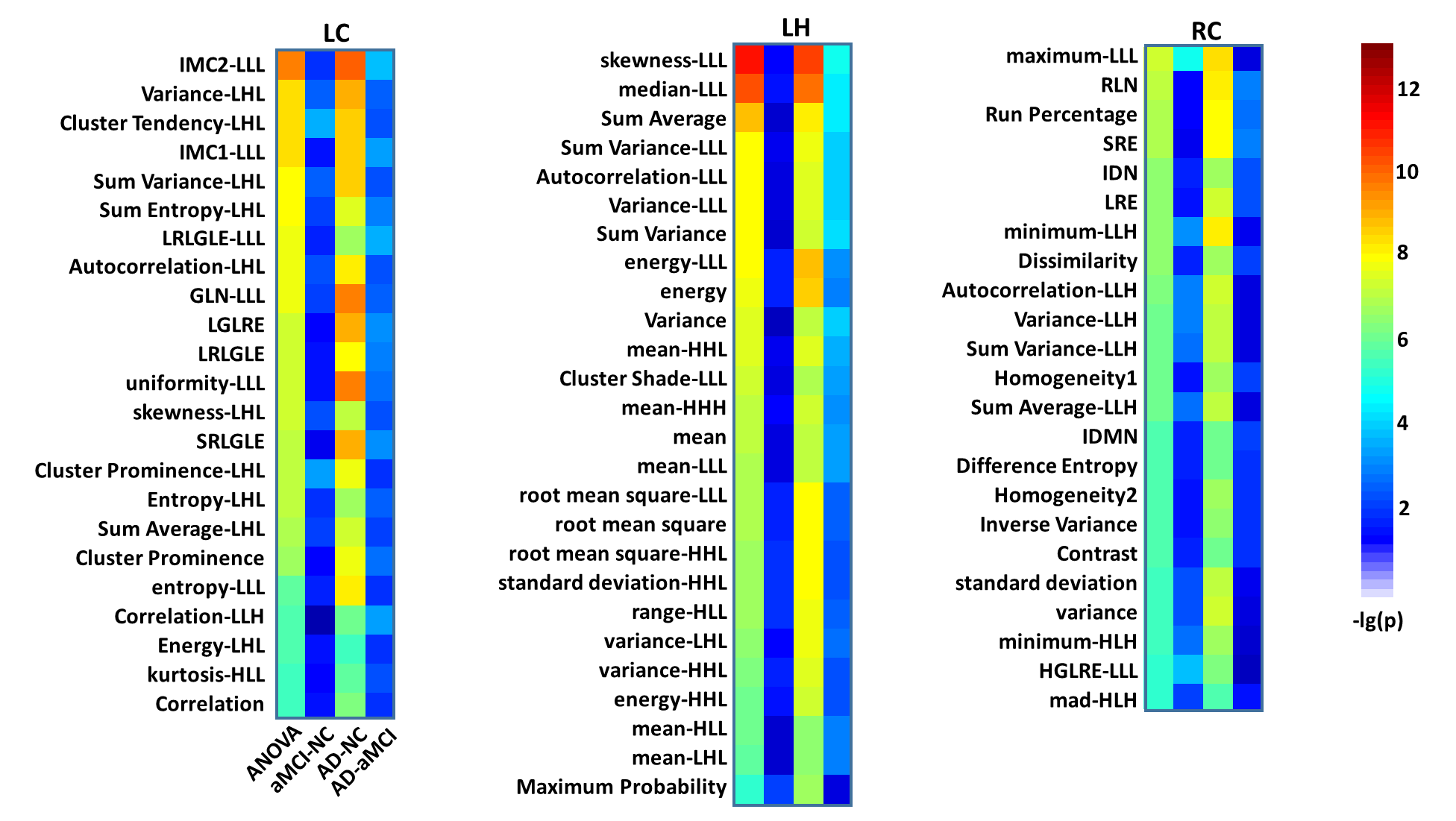
**

**Figure S1.** Heat maps for radiomic features with statistical difference in one hippocampal subregion in discovery data.

A total of 72 radiomic features had statistical difference in one hippocampal subregion, containing LC, LH and RC parts. For each feature in three subregions, there were four grids (P values for ANOVA, t test between aMCI and NC, AD and NC, AD and aMCI respectively), the color bar of which represents the values of -lg(P).

Abbreviations: LC, Left caudal; LH, Left head; RC, Right caudal; IMC2, Informational measure of correlation 2; IMC1, Informational measure of correlation 1; LRLGLE, Long run low gray level emphasis; GLN, Gray level non-uniformity; LGLRE, Low gray level run emphasis; SRLGLE, Short run low gray level emphasis; RLN, Run Length Non-uniformity; SRE, Short run emphasis; IDN, Inverse difference normalized; LRE, Long run emphasis; IDMN, Inverse difference moment normalized; HGLRE, High gray level run emphasis; mad, mean absolute deviation

## Figure S2

**
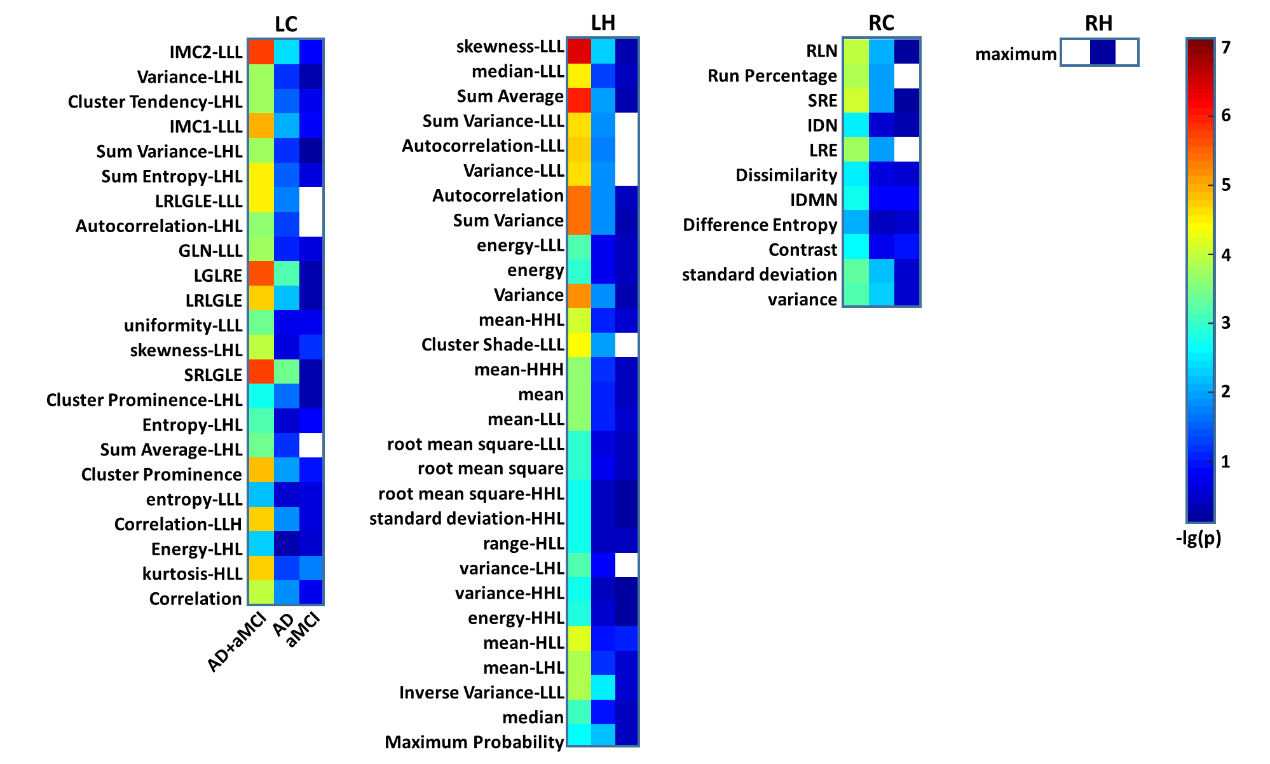
**

**Figure S2.** Heat maps for radiomic features correlating with MMSE in one hippocampal subregion in discovery data.

A total of 64 radiomic features had correlation with MMSE in one hippocampal subregion, containing LC, LH, RC and RH parts. Most of the features were in LC and LH subregions. The color of every grid represents the values of -lg(P), the blank grid means that there is no significant correlation between the related radiomic feature with MMSE in aMCI.

Abbreviations: LC, Left caudal; LH, Left head; RC, Right caudal; RH, Right head; IMC2, Informational measure of correlation 2; IMC1, Informational measure of correlation 1; LRLGLE, Long run low gray level emphasis; GLN, Gray level non-uniformity; LGLRE, Low gray level run emphasis; SRLGLE, Short run low gray level emphasis; RLN, Run Length Non-uniformity; SRE, Short run emphasis; IDN, Inverse difference normalized; LRE, Long run emphasis; IDMN, Inverse difference moment normalized;

## Figure S3


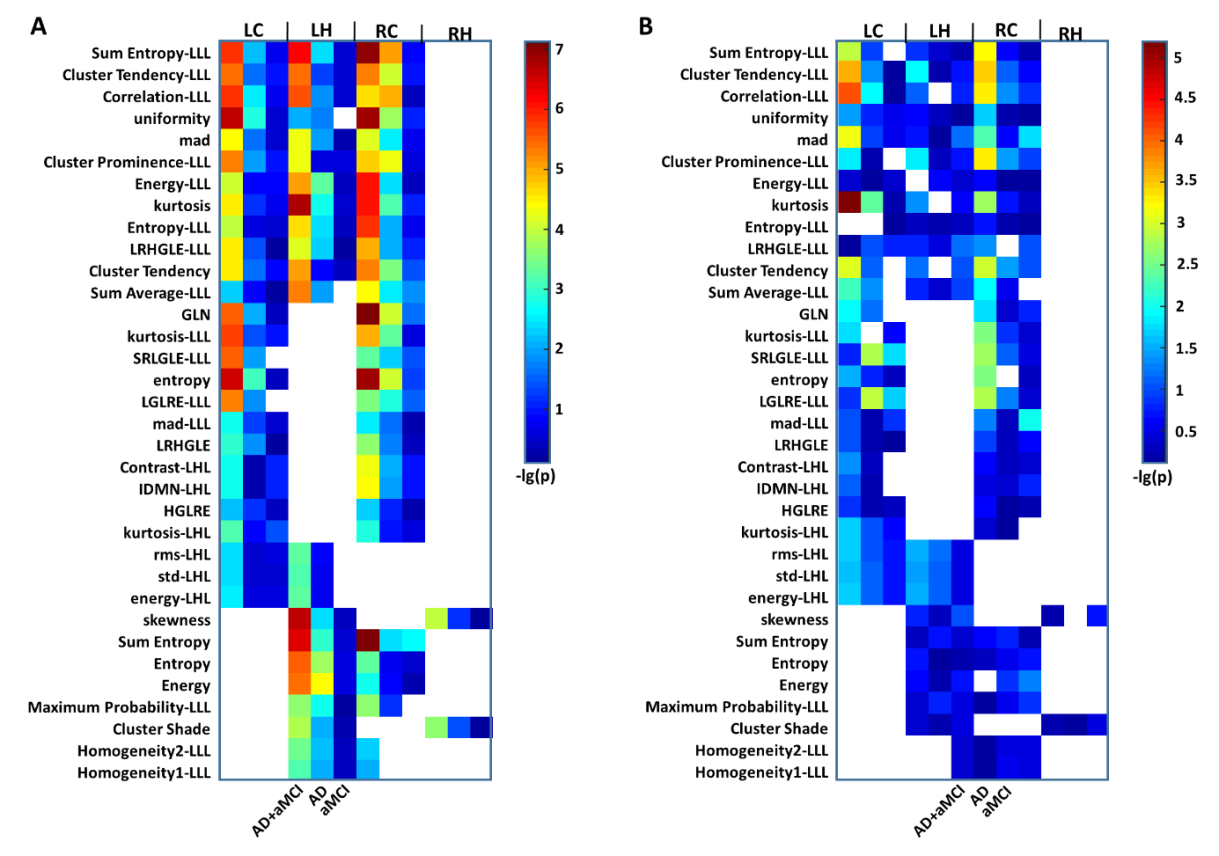


**Figure S3.** Heat maps for radiomic features correlating with MMSE in more than one hippocampal subregion in discovery data and replicated data.

(A) A total of 34 features showed significant correlations with the MMSE in two or three subregions in discovery data. The color bar represents the values of -lg(P), the blank grid means that there is no significant correlation between the related radiomic feature with MMSE in AD and aMCI. (B) The results of replication dataset by using the same procedure.

Abbreviations: LC, Left caudal; LH, Left head; RC, Right caudal; mad, mean absolute deviation; LRHGLE, Long run, high gray level emphasis; GLN, Gray level non-uniformity; SRLGLE, Short run, low gray level emphasis; LGLRE, Low gray level run emphasis; IDMN, Inverse difference moment normalized; HGLRE, High gray level run emphasis

## Figure S4


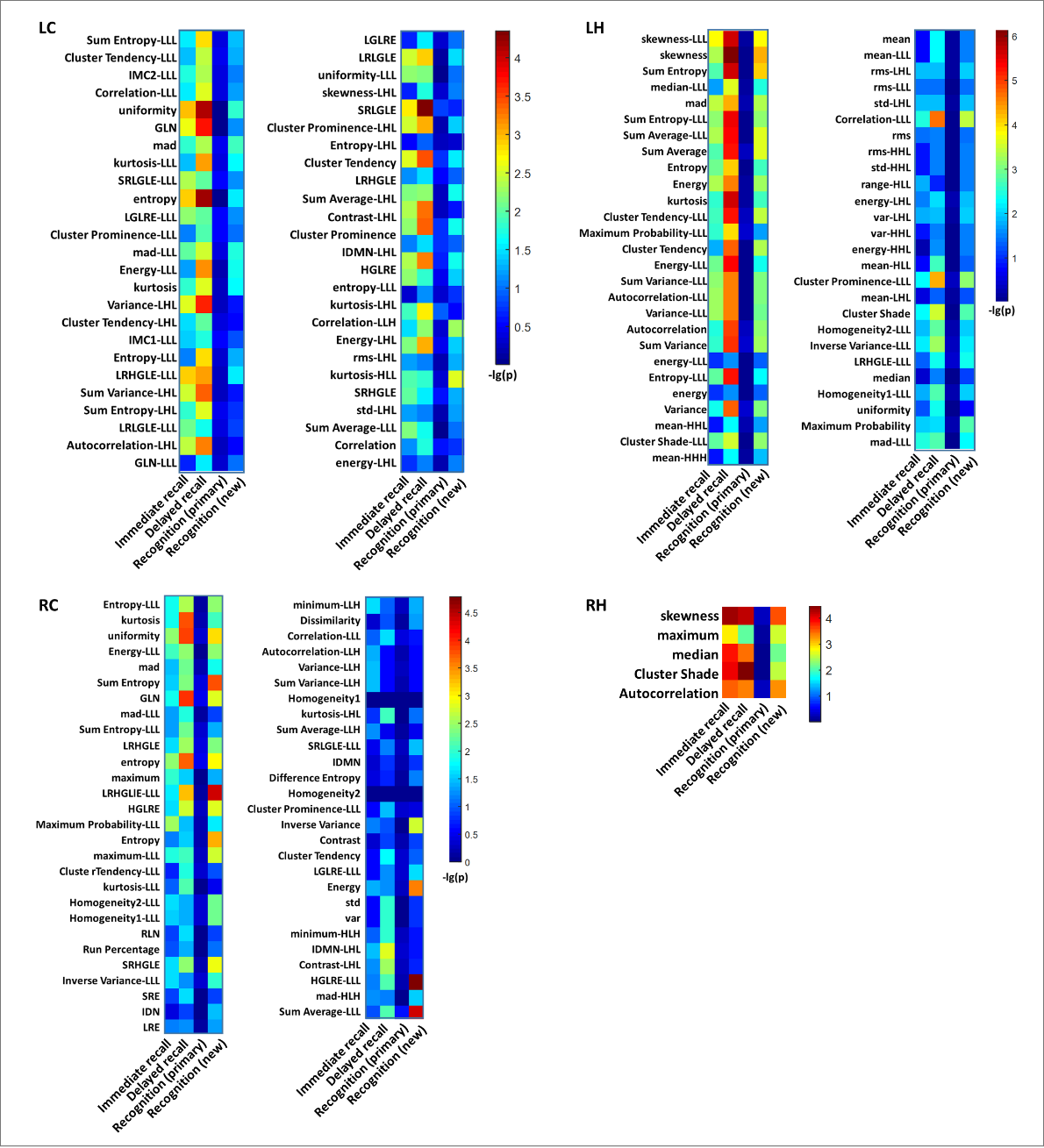


Figure S4. Heat maps for radiomic features correlating with AVLT scores (P < 0.05) in the hippocampal subregions in discovery data.

Abbreviations: LC, Left caudal; LH, Left head; RC, Right caudal; RH, Right head;

# Supplementary Methods

## Cross-validation with leave-four-out for classification AD from NC

In the present study, we used the leave-one-out cross validation (LOOCV) method to evaluate if the radiomic features were good for classification. As suggested by Varoquaux and colleagues ([Varoquaux, 2017](#_ENREF_2); [Varoquaux et al., 2017](#_ENREF_3)), the LOOCV might have potentially overestimated the performance. Hence, we performed the leave-four-out cross-validation (for small sample sizes in the present study) and simulated 1000 times to reevaluate the classification performance, and the results showed that the AUC = 0.91 (ACC=83.55%, SPE=84.66%, SEN=82.66%) for classification AD from NC (Figure S5).


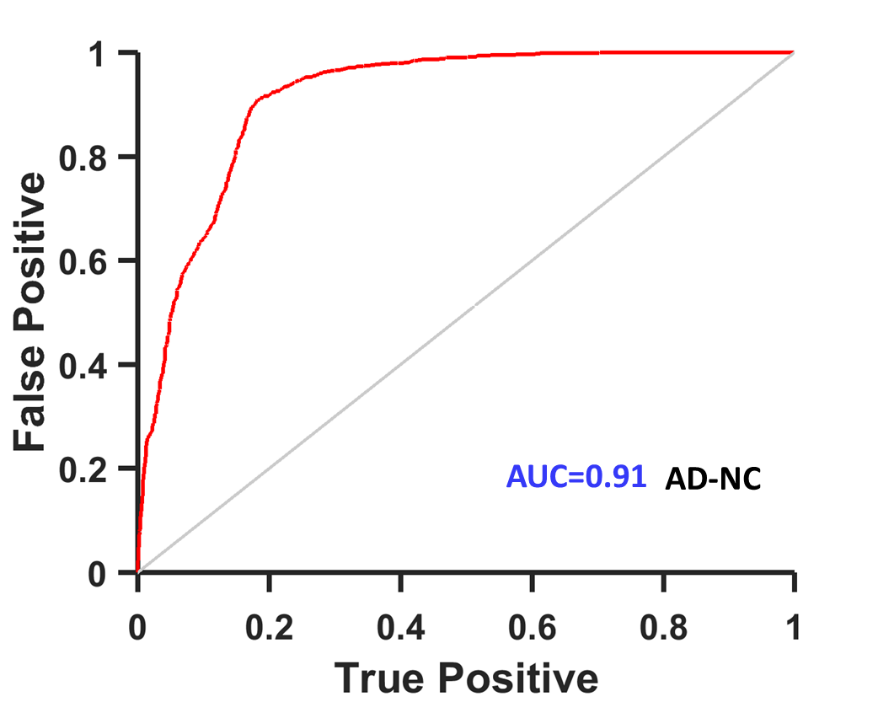


Figure S5. ROC curve for classify AD from NC with leave-four-out cross validation for 1000 times.

## Radiomics features

A total number of 423 MRI imaging features, including intensity, textural and wavelet groups, were extracted in the present study ([Aerts et al., 2014](#_ENREF_1)). The details of these measurements are listed as follows to maintain the scientific integrity of the present study

### 1. Intensity features

**Table S2** Intensity features describe the distribution of voxel intensities within the MRI image through commonly used and basic metrics.

|  | Image feature | | Equation | | Definition |
| --- | --- | --- | --- | --- | --- |
| Intensity features (14) | energy | $\sum_{i}^{N} {X(i)}^{2}$ | | measure of randomness of intensity values in an image | |
|  | entropy | $\sum_{i=1}^{N_{l}} P\left( i \right)\log_{2}P\left( i \right)$ | | represents irregularity of intensity value distribution | |
|  | kurtosis | $\frac{\frac{1}{N}\sum_{i=1}^{N} \left( X\left( i \right)-\bar{X} \right)^{4}}{\left( \sqrt{\frac{1}{N}\sum_{i=1}^{N} \left( X\left( i \right)-\bar{X} \right)^{2}} \right)^{2}}$ | | the peakedness of the histogram or indication of histogram flatness | |
|  | maximum | maximum intensity value of X | |  | |
|  | mean | $\frac{1}{N}\sum_{i}^{N} X(i)$ | | average intensity value of the pixels within the region of interest | |
|  | mean absolute deviation (mad) | mean of the absolute deviations of all voxel intensities around the mean intensity value | | a measure of how much the gray levels differ from the mean | |
|  | median | median intensity value of X | |  | |
|  | minimum | minimum intensity value of X | |  | |
|  | range | range of intensity values of X | |  | |
|  | root mean square (rms) | $\sqrt{\frac{{\sum_{i}^{N} X(i)}^{2}}{N}}$ | |  | |
|  | skewness | $\frac{\frac{1}{N}\sum_{i=1}^{N} {(X\left( i \right)-\bar{X})}^{3}}{\left( \sqrt{\frac{1}{N}\sum_{i=1}^{N} {(X\left( i \right)-\bar{X})}^{2}} \right)^{3}}$ | | symmetry of intensity values in an image | |
|  | standard deviation | $\left( \frac{1}{N-1}\sum_{i=1}^{N} \left( X\left( i \right)-\bar{X} \right)^{2} \right)^{1/2}$ | | a measure of how much variation or dispersion exists from the mean value | |
|  | uniformity | $\sum_{i=1}^{N_{l}} {P(i)}^{2}$ | | measures the homogeneity of the intensity value distribution in an image | |
|  | variance | $\frac{1}{N-1}\sum_{i=1}^{N} \left( X\left( i \right)-\bar{X} \right)^{2}$ | | the spread or variation around the mean (Sum of squares) | |

**X** denotes the three dimensional image matrix. ***N*** voxels. **P** is the first order histogram with ***N_l_*** discrete intensity levels.

$\bar{X}$ is the mean of x.

### 2. Textural features

**Table S3** Textural features describe the patterns or spatial distribution of voxel intensities, which were calculated from a gray level co-occurrence matrix (GLCM) and a gray level run-length matrix (GLRLM), respectively.

|  | Image feature | Equation | Definition |
| --- | --- | --- | --- |
| GLCM based textural features (22) | Autocorrelation | $\sum_{i=1}^{N_{g}} \sum_{j=1}^{N_{g}} ijP(i,j)$ |  |
|  | Cluster Prominence | $\sum_{i=1}^{N_{g}} \sum_{j=1}^{N_{g}} \left[ i+j-\mu_{x}\left( i \right)-\mu_{y}\left( j \right) \right]^{4}P(i,j)$ |  |
|  | Cluster Shade | $\sum_{i=1}^{N_{g}} \sum_{j=1}^{N_{g}} \left[ i+j-\mu_{x}\left( i \right)-\mu_{y}\left( j \right) \right]^{3}P(i,j)$ |  |
|  | Cluster Tendency | $\sum_{i=1}^{N_{g}} \sum_{j=1}^{N_{g}} \left[ i+j-\mu_{x}\left( i \right)-\mu_{y}\left( j \right) \right]^{2}P(i,j)$ |  |
|  | Contrast | $\sum_{i=1}^{N_{g}} \sum_{j=1}^{N_{g}} \left\vert i-j \right\vert^{2}P(i,j)$ | measures the amount of local variation in intensity values |
|  | Correlation | $\frac{\sum_{i=1}^{N_{g}} \sum_{j=1}^{N_{g}} ijP\left( i,j \right)-\mu_{i}(i)\mu_{j}(j)}{\sigma_{x}\left( i \right)\sigma_{y}(j)}$ | measures the linear dependencies of intensity values in an image |
|  | Difference Entropy | $\sum_{i=0}^{N_{g}-1} P_{x-y}\left( i \right){log}_{2}\left[ P_{x-y}(i) \right]$ |  |
|  | Dissimilarity | $\sum_{i=1}^{N_{g}} \sum_{j=1}^{N_{g}} \left\vert i-j \right\vert P(i,j)$ |  |
|  | Energy | $\sum_{i=1}^{N_{g}} \sum_{j=1}^{N_{g}} \left[ P(i,j) \right]^{2}$ |  |
|  | Entropy | $-\sum_{i=1}^{N_{g}} \sum_{j=1}^{N_{g}} P\left( i,j \right){log}_{2}\left[ P\left( i,j \right) \right]$ |  |
|  | Homogeneity1 | $\sum_{i=1}^{N_{g}} \sum_{j=1}^{N_{g}} \frac{P\left( i,j \right)}{1+\left\vert i-j \right\vert}$ | measures the homogeneity of the intensity values of the pixel pair |
|  | Homogeneity2 | $\sum_{i=1}^{N_{g}} \sum_{j=1}^{N_{g}} \frac{P\left( i,j \right)}{1+\left\vert i-j \right\vert^{2}}$ | measures the homogeneity of the intensity values of the pixel pair |
|  | Informational Measure of Correlation 1 (IMC1) | $\frac{HXY-HXY1}{max\left\{ HX,HY \right\}}$ |  |
|  | Informational Measure of Correlation 2 (IMC2) | $\sqrt{1-e^{-2\left( HXY2-HXY \right)}}$ |  |
|  | Inverse Difference Moment Normalized (IDMN) | $\sum_{i=1}^{N_{g}} \sum_{j=1}^{N_{g}} \frac{P\left( i,j \right)}{1+\left( \frac{\left\vert i-j \right\vert^{2}}{N^{2}} \right)}$ |  |
|  | Inverse Difference Normalized (IDN) | $\sum_{i=1}^{N_{g}} \sum_{j=1}^{N_{g}} \frac{P\left( i,j \right)}{1+\left( \frac{\left\vert i-j \right\vert}{N} \right)}$ |  |
|  | Inverse Variance | $\sum_{i=1}^{N_{g}} \sum_{j=1}^{N_{g}} \frac{P\left( i,j \right)}{\left\vert i-j \right\vert^{2}} , i\neq j$ |  |
|  | Maximum Probability | max$\left\{ P\left( i,j \right) \right\}$ |  |
|  | Sum Average | $\sum_{i=2}^{{2N}_{g}} \left[ iP_{x+y}(i) \right]$ |  |
|  | Sum Entropy | $-\sum_{i=2}^{{2N}_{g}} P_{x+y}(i){log}_{2}\left[ P_{x+y}(i) \right]$ |  |
|  | Sum Variance | $\sum_{i=2}^{{2N}_{g}} \left( i-SE \right)^{2}P_{x+y}(i)$ |  |
|  | Variance | $\sum_{i=1}^{N_{g}} \sum_{j=1}^{N_{g}} \left( i-\mu\right)^{2}P(i,j)$ |  |

$P(i,j)$ is the co-occurrence matrix for an arbitrary $\delta$ and$\alpha$

$N_{g}$is the number of discrete intensity levels in the image

μ is the mean of $P(i,j)$

$p_{x}(i)$=$\sum_{j=1}^{N_{g}} P\left( i,j \right)$ is the marginal row probabilities

$p_{y}(i)$=$\sum_{i=1}^{N_{g}} P\left( i,j \right)$ is the marginal column probabilities

$\mu_{x}$ is the mean of$p_{x}$ $\mu_{y}$ is the mean of$p_{y}$

$\sigma_{x}$is the standard deviation of$p_{x}$ $\sigma_{y}$is the standard deviation of$p_{y}$

$p_{x+y}\left( k \right)$=$\sum_{i=1}^{N_{g}} \sum_{j=1}^{N_{g}} P\left( i,j \right)$, i$+$j=k, k=2,3,…,2$N_{g}$

$P_{x-y}\left( k \right)$=$\sum_{i=1}^{N_{g}} \sum_{j=1}^{N_{g}} P\left( i,j \right)$,$\left| i-j \right|$=k, k=0,1,…,$N_{g}-$1

HX=$-\sum_{i=1}^{N_{g}} p_{x}\left( i \right)\log_{2}\left[ p_{x}\left( i \right) \right]$ is the entropy of $p_{x}$

HY=$-\sum_{i=1}^{N_{g}} p_{y}\left( i \right)\log_{2}\left[ p_{y}\left( i \right) \right]$ is the entropy of $p_{y}$

H=$-\sum_{i=1}^{N_{g}} \sum_{j=1}^{N_{g}} P\left( i,j \right)\log_{2}\left[ P\left( i,j \right) \right]$ is the entropy of *P*$\left( i,j \right)$

*HXY1=*$-\sum_{i=1}^{N_{g}} \sum_{j=1}^{N_{g}} P\left( i,j \right)log\left( p_{x}\left( i \right)p_{y}\left( j \right) \right)$

*HXY2=*$-\sum_{i=1}^{N_{g}} \sum_{j=1}^{N_{g}} p_{x}\left( i \right)p_{y}\left( j \right)log\left( p_{x}\left( i \right)p_{y}\left( j \right) \right)$

|  | Image feature | | Equation | | | Definition | | |
| --- | --- | --- | --- | --- | --- | --- | --- | --- |
| GLRLM based textural features (11) | Short Run Emphasis (SRE) | $\frac{\sum_{i=1}^{N_{g}} \sum_{j=1}^{N_{r}} \left[ \frac{p\left( i,j \vert\theta\right)}{j^{2}} \right]}{\sum_{i=1}^{N_{g}} \sum_{j=1}^{N_{r}} p\left( i,j \vert\theta\right)}$ | | | | |  | |
|  | Long Run Emphasis (LRE) | $\frac{\sum_{i=1}^{N_{g}} \sum_{j=1}^{N_{r}} j^{2}p\left( i,j \vert\theta\right)}{\sum_{i=1}^{N_{g}} \sum_{j=1}^{N_{r}} p\left( i,j \vert\theta\right)}$ | | | | |  | |
|  | Gray Level Non-uniformity (GLN) | $\frac{\sum_{i=1}^{N_{g}} \left[ \sum_{j=1}^{N_{r}} p\left( i,j \vert\theta\right) \right]^{2}}{\sum_{i=1}^{N_{g}} \sum_{j=1}^{N_{r}} p\left( i,j \vert\theta\right)}$ | | | | | represents the similarity of intensity values in an image | |
|  | Run Length Non-uniformity (RLN) | $\frac{\sum_{j=1}^{N_{r}} \left[ \sum_{i=1}^{N_{g}} p\left( i,j \vert\theta\right) \right]^{2}}{\sum_{i=1}^{N_{g}} \sum_{j=1}^{N_{r}} p\left( i,j \vert\theta\right)}$ | | | | | measures the run length similarity | |
|  | Run Percentage (RP) | $\sum_{i=1}^{N_{g}} \sum_{j=1}^{N_{r}} \frac{p\left( i,j \vert\theta\right)}{N_{p}}$ | | | | | ratio of the total number of runs to the total number of possible runs measuring the homogeneity of runs. | |
|  | Low Gray Level Run Emphasis (LGLRE) | $\frac{\sum_{i=1}^{N_{g}} \sum_{j=1}^{N_{r}} \left[ \frac{p\left( i,j \vert\theta\right)}{i^{2}} \right]}{\sum_{i=1}^{N_{g}} \sum_{j=1}^{N_{r}} p\left( i,j \vert\theta\right)}$ | | |  | | | |
|  | High Gray Level Run Emphasis (HGLRE) | $\frac{\sum_{i=1}^{N_{g}} \sum_{j=1}^{N_{r}} i^{2}p\left( i,j \vert\theta\right)}{\sum_{i=1}^{N_{g}} \sum_{j=1}^{N_{r}} p\left( i,j \vert\theta\right)}$ | | |  | | | |
|  | Short Run Low Gray Level Emphasis (SRLGLE) | $\frac{\sum_{i=1}^{N_{g}} \sum_{j=1}^{N_{r}} \left[ \frac{p\left( i,j \vert\theta\right)}{i^{2}j^{2}} \right]}{\sum_{i=1}^{N_{g}} \sum_{j=1}^{N_{r}} p\left( i,j \vert\theta\right)}$ | | |  | | | |
|  | Short Run High Gray Level Emphasis (SRHGLE) | $\frac{\sum_{i=1}^{N_{g}} \sum_{j=1}^{N_{r}} \left[ \frac{p\left( i,j \vert\theta\right)i^{2}}{j^{2}} \right]}{\sum_{i=1}^{N_{g}} \sum_{j=1}^{N_{r}} p\left( i,j \vert\theta\right)}$ | | |  | | | |
|  | Long Run Low Gray Level Emphasis (LRLGLE) | $\frac{\sum_{i=1}^{N_{g}} \sum_{j=1}^{N_{r}} \left[ \frac{p\left( i,j \vert\theta\right)j^{2}}{i^{2}} \right]}{\sum_{i=1}^{N_{g}} \sum_{j=1}^{N_{r}} p\left( i,j \vert\theta\right)}$ | |  | | | |  |
|  | Long Run High Gray Level Emphasis (LRHGLE) | $\frac{\sum_{i=1}^{N_{g}} \sum_{j=1}^{N_{r}} p\left( i,j \vert\theta\right)i^{2}j^{2}}{\sum_{i=1}^{N_{g}} \sum_{j=1}^{N_{r}} p\left( i,j \vert\theta\right)}$ | | |  | | | |

$p\left( i,j | \theta\right)$ is the $\left( i,j \right)$th entry in the given run-length matrix $p$ for a direction $\theta$

*N_g_* is the number of discrete intensity values in the image

*N_r_*  is the number of different run lengths

*N_p_* is the number of voxels in the image

### 3. Wavelet features

Wavelet transformation was performed of each hippocampal subregion images in eight directions (LLL, LLH, LHL, LHH, HLL, HLH, HHL, HHH), which resulted 376 wavelet features on the basis of the above 14 intensity features and 33 textural features.


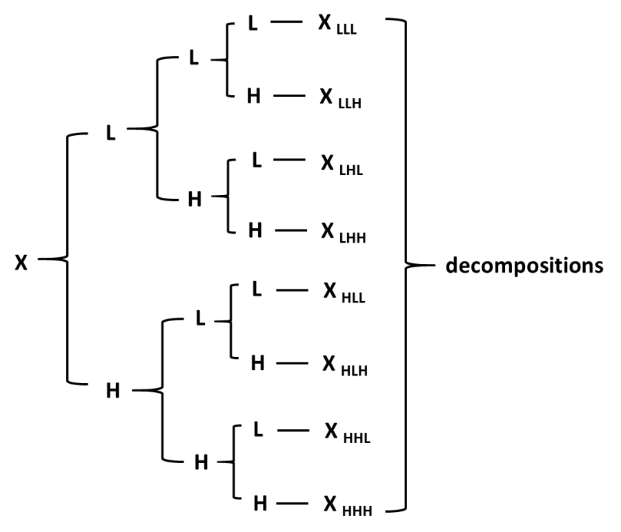


Figure S6. Sketch of the wavelet transform.

# References

Aerts, H.J., et al., 2014. Decoding tumour phenotype by noninvasive imaging using a quantitative radiomics approach. Nat Commun. 5**,** 4006.

Varoquaux, G., 2017. Cross-validation failure: Small sample sizes lead to large error bars. Neuroimage.

Varoquaux, G., et al., 2017. Assessing and tuning brain decoders: Cross-validation, caveats, and guidelines. Neuroimage. 145**,** 166-179.
